# Epigenetic Signatures Discriminate Patients With Primary Sclerosing Cholangitis and Ulcerative Colitis From Patients With Ulcerative Colitis

**DOI:** 10.3389/fimmu.2022.840935

**Published:** 2022-03-16

**Authors:** Manon de Krijger, Ishtu L. Hageman, Andrew Y. F. Li Yim, Jan Verhoeff, Juan J. Garcia Vallejo, Patricia H. P. van Hamersveld, Evgeni Levin, Theodorus B. M. Hakvoort, Manon E. Wildenberg, Peter Henneman, Cyriel Y. Ponsioen, Wouter J. de Jonge

**Affiliations:** ^1^Tytgat Institute for Liver and Intestinal Research, Amsterdam Gastroenterology Endocrinology Metabolism, Amsterdam University Medical Centers, University of Amsterdam, Amsterdam, Netherlands; ^2^Department of Gastroenterology and Hepatology, Amsterdam University Medical Centers, University of Amsterdam, Amsterdam, Netherlands; ^3^Department of Clinical Genetics, Genome Diagnostics Laboratory, Amsterdam Reproduction and Development, Amsterdam University Medical Centers, University of Amsterdam, Amsterdam, Netherlands; ^4^Department of Molecular Cell Biology and Immunology, Amsterdam Infection & Immunity and Cancer Center Amsterdam, Amsterdam University Medical Centers, Free University of Amsterdam, Amsterdam, Netherlands; ^5^Department of Vascular Medicine, Amsterdam University Medical Centers, University of Amsterdam, Amsterdam, Netherlands; ^6^Horaizon BV, Delft, Netherlands; ^7^Department of Surgery, University Clinic of Bonn, Bonn, Germany

**Keywords:** primary sclerosing cholangitis, ulcerative colitis, DNA methylation/methylome, peripheral blood, 850k methylation array, mass cytometry

## Abstract

**Background:**

Primary sclerosing cholangitis (PSC) is a chronic inflammatory liver disease affecting the intra- and extrahepatic bile ducts, and is strongly associated with ulcerative colitis (UC). In this study, we explored the peripheral blood DNA methylome and its immune cell composition in patients with PSC-UC, UC, and healthy controls (HC) with the aim to develop a predictive assay in distinguishing patients with PSC-UC from those with UC alone.

**Methods:**

The peripheral blood DNA methylome of male patients with PSC and concomitant UC, UC and HCs was profiled using the Illumina HumanMethylation Infinium EPIC BeadChip (850K) array. Differentially methylated CpG position (DMP) and region (DMR) analyses were performed alongside gradient boosting classification analyses to discern PSC-UC from UC patients. As observed differences in the DNA methylome could be the result of differences in cellular populations, we additionally employed mass cytometry (CyTOF) to characterize the immune cell compositions.

**Results:**

Genome wide methylation analysis did not reveal large differences between PSC-UC and UC patients nor HCs. Nonetheless, using gradient boosting we were capable of discerning PSC-UC from UC with an area under the receiver operator curve (AUROC) of 0.80. Four CpG sites annotated to the *NINJ2* gene were found to strongly contribute to the predictive performance. While CyTOF analyses corroborated the largely similar blood cell composition among patients with PSC-UC, UC and HC, a higher abundance of myeloid cells was observed in UC compared to PSC-UC patients.

**Conclusion:**

DNA methylation enables discerning PSC-UC from UC patients, with a potential for biomarker development.

## Introduction

Primary sclerosing cholangitis (PSC) is a chronic cholestatic liver disease, characterized by inflammation and fibrosis of the intra- and extrahepatic bile ducts. The etiology of PSC is largely unknown and there is still no medical treatment with a proven benefit on disease progression (1). The male to female ratio is 2:1, and interestingly, up to 80% of PSC patients has concomitant inflammatory bowel disease (IBD), of which the majority has ulcerative colitis (UC) (2–4). Comparing patients with UC and patients with PSC-UC could give more insights in pathophysiological processes underlying PSC.

The co-occurrence of PSC and IBD has led to various hypotheses linking these two disease entities. One of these hypotheses is the aberrant gut-homing lymphocyte paradigm, which hypothesizes that circulating T-lymphocytes that are primed in the gut and express gut-homing molecules integrin α4β7 and C-Chemokine Receptor 9 (CCR9), can infiltrate in the liver *via* aberrantly expressed adhesion molecules in PSC-affected liver (5, 6). This mechanism may be due to genetic predisposition of the host and/or epigenetic changes in circulating lymphocytes.

Over the past few years, several genome-wide association studies (GWAS) in PSC have identified multiple susceptibility loci for PSC-IBD, with none of the loci being associated with this gut-lymphocyte homing paradigm. Interestingly, PSC-IBD patients and IBD patients share only a limited number of risk loci, suggesting that the combination of PSC-IBD comprises a distinct disease manifestation (7–9). Nevertheless, genetic variation alone cannot account for total disease liability in PSC-IBD, emphasizing the role of internal and external exposures (the “exposome”) including epigenetic factors that may contribute to the disease (10).

Epigenetics comprises heritable processes that involve transcriptional regulation without changing the nucleotide sequence. One of the most studied epigenetic marks is DNA methylation, which represents the addition of a methyl to a base, typically a cytosine that is directly followed by a guanine (CpG). DNA methylation in the promotor region of a gene has been inversely associated with gene expression and is thought to prevent binding of transcription factors, thereby silencing gene expression (11, 12). Differences in DNA methylation of blood cells has been described previously in the context of IBD, where patients with Crohn’s disease (CD) differed from patients with UC (13) and healthy controls (14). Similar observations have been made in colonic tissue of IBD patients (13). Furthermore, patients with PSC with and without IBD share common methylation differences compared to controls (15). Recently, it was reported that in patients with PSC the DNA methylation age, as estimated using the so-called Horvath clock, was higher than the chronological age (16, 17). This age acceleration has proven to have predictive properties regarding disease activity in different diseases, both based on peripheral blood as well as, amongst others, in liver tissue (16–18).

In this study, we hypothesized that the peripheral blood DNA methylome of patients with concomitant PSC-UC is distinct from that of patients with UC without PSC and healthy controls (HC). Accordingly, we investigated the DNA methylome and performed supervised classification analyses to see whether we could accurately discern PSC-UC from UC and identify CpG loci that contributed to this classification. To ascertain that discriminating differences in DNA methylation between PSC-UC and UC were not solely the result of underlying differences in cellular composition in these different disease states, we also performed mass cytometry to characterize the cellular heterogeneity.

## Materials and Methods

### Patients

Patients included in the current study were selected from a well-characterized cohort of the ‘Epi PSC PBC project’, a large population-based cohort to study patients with cholestatic liver diseases [PSC and primary biliary cholangitis (PBC)] as well as IBD patients in the Netherlands (2). Patients with prior liver transplantation, colorectal carcinoma, cholangiocarcinoma or prior bowel surgeries were excluded. Out of 1183 cases, 18 patients with PSC-UC, 17 patients with UC, as well as 12 healthy controls (HC) were selected for DNA methylation analysis. Only male patients were included, and groups were matched for age, UC and medication use (**Table 1**).

**Table 1:** Baseline characteristics.

	Cohort 1				Cohort 2			
	PSC-UC (n=17)	UC (n=17)	Healthycontrols (n=12)	p-value	PSC-UC (n=10)	UC (n=10)	Healthy controls (n=10)	p-value
**Male [n (%)]**	17 (100)	17 (100)	12 (100)	–	10 (100)	10 (100)	10 (100)	–
**Age [median (IQR)]**	41 (30-55)	34 (31-54)	39 (29-50)	0.911	40 (35-58)	58 (49-62)	30 (28-53)	0.022
**Age at diagnosis PSC [median (IQR)]**	33 (24-46)	–	–	–	33 (25-37)	–	–	–
**Duration PSC in years [median (IQR)]**	5 (2-12)	–	–	–	7 (6-15)	–	–	–
**Age at diagnosis UC [median (IQR)]**	27 (20-37)	29 (19-40)	–	0.865	35 (22-39)	30 (19-46)	–	0.796
**Duration UC in years [median (IQR)]**	16 (7-21)	11 (5-15)	–	0.218	9 (5-19)	22 (14-34)	–	0.019
**Medication use [n (%)]**								
** UDCA**	17 (100)	0 (0)	–	0.000	8 (80)	0 (0)	–	0.001
** Anti-TNF-α**	0 (0)	0 (0)	–	–	0 (0)	0 (0)	–	–
** Mesalazine**	17 (100)	17 (100)	–	–	9 (90)	9 (90)	–	1.000
** Thiopurines**	8 (47)	9 (53)	–	1.000	0 (0)	1 (10)	–	1.000
**Montreal classification [n (%)]**				0.144				0.164
** Pancolitis**	15 (88)	10 (59)	–		7 (70)	6 (60)	–	
** Left sided**	2 (12)	5 (29)	–		1 (10)	4 (40)	–	
** Proctitis**	0 (0)	1 (6)	–		2 (20)	0 (0)	–	

For continuous variables the Kruskal Wallis test was used for comparing 3 groups, whereas the Mann Whitney-U test was used for comparing 2 groups. For dichotomous variables, Fisher’s Exact test was used. P-value < 0.05 was considered statistically significant. PSC, primary sclerosing cholangitis; UC, ulcerative colitis; IQR, interquartile range; UDCA, Ursodeoxycholic acid.

For mass cytometry analysis, peripheral blood was collected from 10 patients with PSC-UC, 10 patients with UC and 9 healthy volunteers (**Table 1**). Again, only male patients were included, and biological use was an exclusion criterion to minimize influences on immunological cell distribution. Both protocols were approved by the Medical Ethics Committee at the Amsterdam UMC, University of Amsterdam (METC 06–267/E and METC 2018-050). All samples were collected with written informed consent.

### DNA Isolation and *In Vitro* DNA Methylation Analysis

Genomic DNA was extracted from whole peripheral blood samples using an Autopure LS system (Qiagen, according to the manufacturer protocol) and stored at 4°C. Genomic DNA was sent to GenomeScan (Leiden) for bisulfite conversion and DNA methylation profiling. In short, bisulfite converts unmethylated cytosines into uracil, whereas the methylated cytosines remain unchanged. DNA methylation profiling was performed using the Illumina HumanMethylation Infinium EPIC BeadChip (850K) array, yielding the methylation status of approximately 850.000 CpG sites (19). Samples were randomized across the different chip slides to reduce possible batch effects.

### *In Silico* DNA Methylation Analysis

Raw microarray data was imported in R statistical programming environment (v4.0.2) using the Bioconductor (v3.11) package *minfi* (v1.32.0) (20). Quality control was performed using *MethylAid* (v1.22.0) (21) and *shinyMethyl* (v1.24.0) (22), suggesting no apparent technical issues, such as slide-related batch effects. The raw data was then normalized either through functional normalization for differential methylation analyses using *limma* (v3.44.3) (23), or through noob for classification analyses (24). Preprocessing of the data consisted of removing probes that were associated to known genetic variants and cross-reactive probes (25). Allosome-associated probes were not specifically filtered out in the preprocessing steps as the cohort consisted of only males. Repetitive element-binding probes were not excluded as their effect on the percentage methylation was found to be minimal (26). Quality control based on principal component analysis resulted in the removal of one sample with PSC-UC due to its outlier status. Differential methylation analyses using *limma* were performed using the following design matrix:


methylation∼age+steroids+UC:PSC,

where we compared PSC-UC with UC and PSC-UC with HC. The resultant p-values were adjusted for multiple testing using the Benjamini–Hochberg method. For the hypothesis-driven approach we extracted all probes of length *N_gene_* associated to a particular gene of interest and calculated the aggregated p-value using the Fisher method. Next, we constructed a null distribution of p-values by aggregating p-values calculated from 5000 randomly selected stretches of *N_gene_* consecutive of probes thereby capturing the correlated nature of DNA methylation that occurs in a particular region. By comparing the observed aggregated p-value with that of the null distribution, we obtained the final p-value. Visualizations were generated using *ggplot2* (v3.3.2) (27) and *ggbio* (v1.36.0) (28).

### DNA Methylation Acceleration Analysis

The DNA methylation age in years was calculated using the Horvath clock as implemented in *wateRmelon* (v2.0.0) (17, 29). The difference between the DNA methylation age and the chronological age called the age acceleration.

### DNA Methylation Blood Cell Estimation

The blood cell distribution as estimated from the DNA methylation data using the estimateCellCounts2 function as implemented in the *FlowSorted.Blood.EPIC* (v1.12.1) package (30). In short, this package estimates the cellular composition per sample using a quadratic programming approach. Resultant estimates were subsequently compared between groups using a two-way ANOVA test as implemented in R.

### Gradient Boosting Analysis

Gradient boosting analysis was used to classify patients with PSC-UC from patients with UC. To identify the CpGs that contributed the most to the predictive performance, covered information disentanglement (CID) was implemented (31–34). In short, data was split up in a train (2/3) and test (1/3) set, whereupon the classifier was trained through repeated cross-validation on the training set. The performance of the resulting model was subsequently evaluated on the withheld test set. The area under the receiver operating characteristic (AUROC) scores were computed within each repetition of cross-validation and averaged for the final test AUROC. We reported the CID-derived feature importance scores for the CpG sites that contributed the most to the prediction model (31, 32).

### DNA Sequencing NINJ2

DNA sequencing of *NINJ2*-associated loci was performed through Sanger sequencing using the BigDye Terminator v1.1 Cycle Sequencing kit. In short, primers were designed against the region encompassing the CpG loci of interest (**Supplementary Figure 5** and **Supplementary Table 4**). Genomic DNA of all patients included in the DNA methylation data was used as input for specific PCR amplification of the region of interest. A comprehensive overview of the PCR amplification protocol can be found in the supplementary methods. The resultant PCR products were subsequently sequenced at the Core Facility Genomics, Amsterdam UMC. Reads were then aligned using BioEdit and the CpGs of interest were analyzed for variants that might have introduced bases other than cytosine. Seven samples (n=3 PSC-UC and n=4 UC) were excluded after quality control.

### mQTL Database Analysis

The mQTL database was interrogated for the *NINJ2*-associated predictor CpGs to identify the potential relationship with catalogued genetic variants (mQTL; http://www.mqtldb.org/).

### Bisulfite Conversion, PCR and Illumina MiSeq Sequencing

Technical validation of the *NINJ2*-associated loci of interest (cg26654770 and cg14911689) annotated to the *NINJ2* gene was performed through targeted amplicon sequence analysis using the Illumina MiSeq platform. Primers were designed in MethPrimer (35) **(** **Supplementary Table 4**). DNA samples from five PSC-UC, two UC and two HC patients (cohort 1, **Table 1**) were bisulfite converted according to standard protocol using the EZ DNA methylation kit (Zymo Research) (36). Amplicons were made from bisulfite converted DNA PCR and further purified with the Agencourt AMPure PCR purification kit (Beckman Coulter). During a second PCR, amplicons were elongated using TruSEQ indices and Illumina sequence adapters, whereupon they were purified and pooled in stoichiometric amounts. Quality control of the amplicon length within the pools was performed using Agilent 2100 BioAnalyzer. DNA concentrations were measured using Qubit 2.0 Fluorometer (ThermoFisher) and equalized to equimolar concentrations for all subject pools. MiSeq amplicon sequencing was then performed according to the standard protocol. Raw sequence data was mapped, aligned, and analyzed using Bismark (37) and visualized using Integrative Genomics viewer (v 2.3.57) against the bisulfite-converted human genome hg19. A minimum of 120 reads per sample-derived amplicon was deemed successful.

### Quantitative Real-Time Polymerase Chain Reaction

Messenger RNA was extracted from frozen PBMCs using the Bioline ISOLATE II RNA mini kit (GC biotech B.V. Alphen a/d Rijn, the Netherlands) according to manufacturer’s instructions. RNA concentration was measured using the Nanodrop 1000 spectrophotometer (Nanodrop Technologies, Wilmington, DE, USA). cDNA was synthesized using the Revertaid first strand cDNA synthesis kit (Fermentas, St. Leon-Rot, Germany). A quantitative polymerase chain reaction (qPCR) was performed using SensiFAST SYBR No-ROX (GC Biotech B.V.) on a BioRad (CFX96 real-time qPCR thermocycler). Resultant gene expression levels were calculated using LinRegPCR (38). After stability analysis in geNorm, two human reference genes were selected for normalization; Glyceraldehyde 3-Phosphate Dehydrogenase (*GAPDH*) and Hypoxanthine-guanine-fosforibosyl-transferase (*HPRT*) (39). Primers were either obtained by Qiagen or synthesized by Sigma (**Supplementary Table 4**).

### Mass Cytometry

Cryopreserved PBMCs were thawed and washed with medium (RPMI+20% fetal bovine serum (FBS)) whereupon they were resuspended in PBS. For cellular viability assessment, single-cell suspensions were incubated with Cisplatin (5 µM, Fluidigm) for 5 minutes and washed with cell staining buffer (CSB, Fluidigm). Cells were incubated with Human TruStain FcX Fc receptor blocking solution (Biolegend), after which cells were stained with a mix of metal-conjugated antibodies against cell surface markers (**Supplementary Table 5**, ‘pre-fixation’), washed with CSB and fixed with 1.6% PFA. Cells were permeabilized by Maxpar Barcode Perm Buffer (Fluidigm), incubated with mass tag barcodes and stained with the remaining metal-conjugated antibodies (**Supplementary Table 5**, Fluidigm). For intracellular staining, cells were washed with Perm-S buffer (Fluidigm) and incubated with antibodies against CTLA-4 and CES-1 (**Supplementary Table 5**, ‘nuclear staining’), washed and incubated with the corresponding secondary antibodies. Antibodies were fixated with 1.6% PFA/PBS, washed and incubated overnight with ^191/193^Ir DNA intercalator (1:4000) diluted in Fix-and-Perm Buffer (Fluidigm). Acquisition was performed on the Cytometry by time of flight (CyTOF)3-Helios. Sample was diluted in H2O and supplemented with 10% v/v of EQ Four Element Calibration beads (Fluidigm). After acquisition data was normalized and individual files were deconvoluted using the CyTOF software v6.7 functions.

Normalized.fcs files were uploaded in to Cytobank (40) for analysis and quality control. Viable CD45^+^ singlets were selected according to gating strategy previously described (41). Both batches of samples included a technical replicate. Potential batch effects were investigated through manual inspection of marker distributions and overlap in clusterings. Different lineages (B-cells, CD4^+^ T-cells, CD8^+^ T-cells, myeloid cells and NK-cells) were clustered and color-coded using FlowSOM and subsequent manual annotation (42). Data is visualized using viISNE, a visualization tool for high-dimensional single-cell data based on the t-dDistributed Stochastic Neighbor Embedding (t-SNE) algorithm (43).

### Patient Characteristic Statistical Analyses

Patient characteristics are presented as median and interquartile range (IQR; 25th- 75th percentile). Dichotomous variables are presented as percentage (%) of the cohort. Differences were calculated with the chi-square test or Fisher’s exact test for categorical variables. Numerical data were compared using a Mann‐Whitney U test or One-way ANOVA, or a Kruskal‐Wallis test with Dunn’s correction for multiple testing. Statistical analysis was performed in SPSS statistical software for Windows version 26.0 (SPSS, Chicago, USA) or GraphPad Prism 8. A p-value <0.05 was considered statistically significant.

## Results

### The Genome-Wide Methylome of Patients With PSC-UC Is Comparable to That From Patients With UC and Healthy Controls

DNA methylation was investigated in peripheral blood samples from 17 patients with PSC-UC, 17 patients with UC and 12 HCs. The characteristics of the included patients are shown in **Table 1** (cohort 1). To limit the number of variables, we included only male patients, with all groups being matched for age (median age 41, 34 and 39 years for PSC-UC, UC and HC, respectively), UC duration (16 and 11 years for PSC-UC and UC, respectively) and medication use (all patients with PSC-UC and UC used mesalazine, 50% used thiopurins and none used biologicals). Median PSC duration at time of inclusion was 5 years. None of the patients had undergone liver transplantation at the time of sampling.

**Figure 1:**
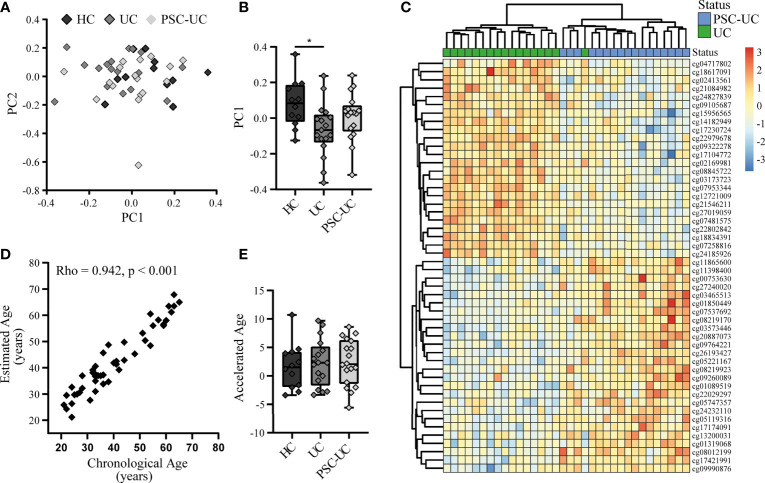
The genome-wide methylome of patients with PSC-UC is comparable to that from patients with UC and healthy controls. **(A)** Principal component (PC) analysis of the methylome of patients with PSC-UC (light grey), UC (grey) and HC (dark grey). **(B)** Association of PC1 with all groups. **(C)** Heatmap of top 50 differentially methylated positions between PSC-UC (blue) and UC (green). **(D)** Correlation between chronological age (years) and estimated DNA methylation age (years) in patients with PSC-UC, UC and HC. **(E)** Comparison of accelerated age (difference between chorological age and estimated age) between patients with PSC-UC, UC and HC. A p-value <0.05 was considered statistically significant (*p < 0.05).

Global methylation analysis through principal component (PC) analysis did not present a clear separation of the samples according to disease status **(** **Figure 1A****)**. Notably, PC1 presented a larger separation between HC and UC (p-value = 0.021), than HC and PSC-UC (p-value = 0.347) or PSC-UC and UC (p-value = 0.292) **(** **Figure 1B**).

Next, we specifically investigated whether any of the probes were differentially methylated when comparing PSC-UC with UC. While we were able to observe differentially methylated positions (DMPs) that were visibly differentially methylated **(** **Figure 1C****)**, they were not statistically significant after correction for multiple testing (**Table 2**). The most differentially methylated probe was cg02169981 (annotated to *WNT11*), which displayed hypermethylation among patients with UC compared to patients with PSC-UC (**Supplementary Figure 1A**). Expanding our search to regions of contiguous differential methylation (DMRs) when comparing either PSC-UC with UC, or PSC-UC with HC yielded no statistically significant differences (**Supplementary Table 1**).

**Table 2:** Differentially methylated positions (DMPs) when comparing PSC-UC with UC.

Location	Associated gene	Chromosome	Difference in methylation in PSC-UC vs UC	p-value	Adj. p-value	Name
cg02169981	*WNT11*	Chr 11	Hypomethylation	2.20E-06	0.770179	Wnt Family Member 11
cg26193427	*INPP5A*	Chr 10	Hypermethylation	5.44E-06	0.770179	Inositol Polyphosphate-5-Phosphatase A
cg07953344	*NRP2*	Chr 2	Hypomethylation	5.59E-06	0.770179	Neuropilin 2
cg17421991	*MNX1*	Chr 7	Hypermethylation	5.80E-06	0.770179	Motor Neuron And Pancreas Homeobox 1
cg08012199	ARHGAP20*	Chr 11	Hypermethylation	6.23E-06	0.770179	Rho GTPase Activating Protein 20
cg27019059	RYK*	Chr 3	Hypomethylation	8.73E-06	0.770179	Receptor Like Tyrosine Kinase
cg13200031	*FAM163A*	Chr 1	Hypermethylation	9.66E-06	0.770179	Family With Sequence Similarity 163 Member A
cg09990876		Chr 1	Hypermethylation	1.04E-05	0.770179	
cg00753630	*ATP6VOE2-AS1*	Chr 7	Hypermethylation	1.18E-05	0.770179	Vacuolar Proton Pump Subunit E 2
cg18617091	*TIMPRSS6*	Chr 22	Hypomethylation	1.36E-05	0.770179	Transmembrane Serine Protease 6

The 10 most differentially methylated positions. *Neighbour gene. P-values were calculated through linear regression using limma and adjusted for multiple testing using the Benjamini–Hochberg method.

Having identified no clear differences in methylation at a genome-wide level, we adopted a hypothesis-driven approach where we interrogated the methylation status of previously reported loci/genes of interest, obtained from previous genome- (GWAS) or transcriptome- wide association studies (TWAS) (7–9, 44–49). When comparing PSC-UC with UC, only two genes were significantly enriched for nominally differentially methylated probes: *BACH2* and *ASAP2* (**Supplementary Table 2** and **Supplementary Figure 2**). Comparing PSC-UC with HC indicated that probes associated with eight genes showed a statistically significant difference (**Supplementary Table 2**). *UBASH3A*, the most significant SNP in a large GWAS study and associated with a lower risk of PSC (8), showed differential methylation centering around the promotor region when comparing PSC-UC with HC (**Supplementary Figure 2C**).

In a recent study, it was reported that patients with PSC presented an increased DNA methylation age relative to their chronological age, which was especially apparent in samples obtained from patients at an advanced disease state (16). Using the same method we estimated the DNA methylation age of all samples and compared the estimated and chronological age (17). Overall, we found that the DNA methylation age correlated well with the chronological age (**Figure 1D**; Spearman’s Rho 0.942, p-value < 0.001). Calculating the difference between the DNA methylation age and the chronical age revealed a median difference of 2.1 years in patients with PSC-UC (range -5.6-8.6), which did not significantly differ from either patients with UC or HCs (median 2.3 (range -3.3-9.7) and 1.5 (range -3.4-10.7), respectively (p-value = 0.874, **Figure 1E**). We note however that the use of different normalization methods resulted in larger or smaller differences between the predicted age and the chronological age, with a more notable deviation when using quantile normalization. However, even with the larger differences from the quantile normalization, we did not identify any significant differences between phenotypes (**Supplementary Figure 3**).

Taken together, we observed no big differences between the three groups at a DNA methylome-wide level, nor did we find any statistically significant differences between DMPs or DMRs of patients with PSC-UC and either patients with UC or healthy controls.

### Classification Analysis Distinguishes PSC-UC From UC

**Figure 2:**
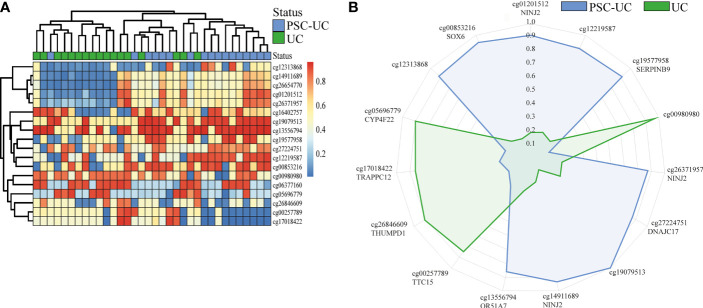
Gradient boosting analysis distinguishes PSC-UC from UC. **(A)** Heatmap of 18 differentially methylated positions contributing to the predictive model distinguishing PSC-UC from UC. **(B)** Radar plot depicting the 15 most predictive CpG loci that are capable of distinguishing patients with PSC-UC from UC. The axes represent the mean scaled changes for the top 15 most discriminative CpG sites.

Next, we investigated whether DNA methylation could be of use for distinguishing patients with PSC-UC from patients with UC without PSC. To this end, we performed a classification analysis using repeated cross-validation with gradient boosting, where we sought to capitalize on potential non-linear relationships between the CpG loci of interest and the presence of PSC-UC (34). Altogether, the classification analysis yielded a predictive model with an area under the receiver-operator characteristic curve (AUROC) of 0.80. Subsequent permutation analyses indicated that 18 CpGs contributed significantly to the model (**Table 3** and **Figure 2A**). Of these 18 CpGs, PSC-UC-associated hypermethylation was observed for *NINJ2*, cg12219587, *SERPINB9*, *DNAJC17*, cg19079513, *OR51A7*, cg12313868 and *SOX6* whereas hypomethylation was found for cg00980980, *TTC15*, *THUMPD1*, *TRAPPC12* and *CYP4F22* (**Figure 2B**).

**Table 3:** Predictor CpG loci capable of distinguishing PSC-UC from UC.

Location	Associated gene	Chromosome	Difference in methylation in PSC-UC vs UC	Feature Importance	Name
cg01201512	*NINJ2*	Chr 12	Hypermethylation	1.442312	Nerve Injury-Induced Protein 2, Ninjurin-2
cg12219587			Hypermethylation	1.295918	
cg19577958	*SERPINB9*	Chr 6	Hypermethylation	1.265306	Serpin Family B Member 9, Serpin B9, Granzyme B inhibitor
cg00980980			Hypomethylation	0.858523	
cg26371957	*NINJ2*	Chr 12	Hypermethylation	0.639473	Nerve Injury-Induced Protein 2, Ninjurin-2
cg27224751	*DNAJC17*	Chr 15	Hypermethylation	0.632653	DnaJ Heat Shock Protein Family (Hsp40) Member C17
cg19079513			Hypermethylation	0.612245	
cg14911689	*NINJ2*	Chr 12	Hypermethylation	0.583521	Nerve Injury-Induced Protein 2, Ninjurin-2
cg13556794	*OR51A7*	Chr 11	Hypermethylation	0.520408	Olfactory Receptor Family 51 Subfamily A Member 7, Odorant receptor
cg00257789	*TRAPPC12/TTC15*	Chr 2	Hypomethylation	0.448323	Trafficking Protein Particle Complex 12
cg26846609	*THUMPD1*	Chr 16	Hypomethylation	0.397421	THUMP domain-containing protein 1
cg17018422	*TRAPPC12/TTC15*	Chr 2	Hypomethylation	0.355172	Trafficking Protein Particle Complex 12
cg05696779	*CYP4F22*	Chr 19	Hypomethylation	0.306122	Cytochrome P450 family 4 subfamily F member 22
cg12313868			Hypermethylation	0.244898	
cg00853216	*SOX6*	Chr 11	Hypermethylation	0.204082	Transcription factor SOX-6
cg26654770	*NINJ2*	Chr 12	Hypermethylation	0.196723	Nerve Injury-Induced Protein 2, Ninjurin-2
cg06377160	*SNX19*	Chr 11	Hypomethylated	0.173469	Sorting nexin-19
cg16402757	*CUL2*	Chr 10	Hypermethylated	0.134404	Cullin-2

The 18 CpG loci returned by gradient boosting that were capable of distinguishing PSC-UC from UC.

**Figure 3:**
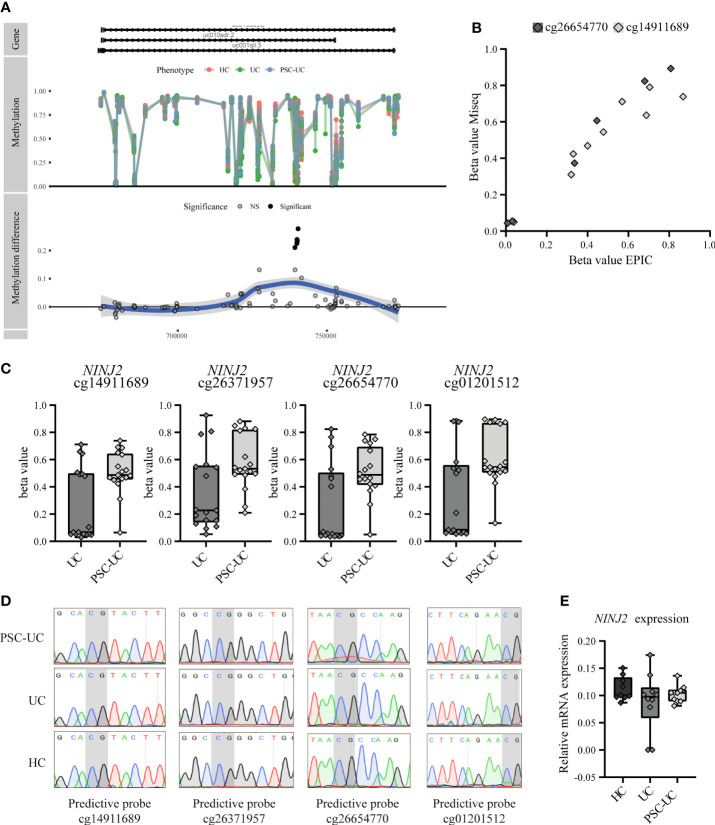
Predictive CpGs annotated to *NINJ2* were all hypermethylated in PSC-UC compared to UC patients. **(A)** Visualization of *NINJ2* by plotting the actual percentage methylation for PSC-UC, UC and HC (“Methylation”) as well as the difference between PSC-UC and UC in percentage methylation (“Methylation difference”) relative to the position on the genome. **(B)** Visual correlation of the percentage methylation observed for cg26654770 and cg14911689 as calculated using the 850k DNA methylation array and through MiSeq sequencing for 5 PSC-UC, 2 UC and 2 HC patients. **(C)** Differences in percentage methylation (beta value) in patients with UC (n = 17) and PSC-UC (n = 17). **(D)** Representative images of Sanger sequencing traces surrounding the CpG loci of interest (marked in grey) that are annotated to *NINJ2* in PSC-UC (n = 14), UC (n = 13) and HCs (n = 12). **(E)** Relative mRNA expression of *NINJ2* normalized to the household genes *GAPDH* and *HPRT* in peripheral blood mononuclear cells of PSC-UC (n = 10), UC (n = 10) and HCs (n = 9).

While most predictive CpGs were embedded in a gene as single CpG, four were found to be all localized within the gene *NINJ2*. All four of the *NINJ2*-associated predictor CpGs were adjacent to one another and were located in an intronic region (**Figure 3A** and **Supplementary Figure 5**). Technical validation through bisulfite sequencing of two of the four predictor CpGs (cg26654770 and cg14911689) confirmed the methylation pattern (**Figure 3B**). Notably, the observed methylation signal of the *NINJ2*-associated DMPs presented itself as a clustered pattern around 0%, 50% and 100% methylation (**Figure 3C**), a pattern characteristic of underlying genetic variants (50). However, sequencing the underlying region of interest revealed no single nucleotide polymorphism (SNP) or other local genetic variants at the loci of interest (**Figure 3D**). As more distal genetic variants might affect DNA methylation, which have been termed methylation quantitative trait loci (mQTL), we interrogated the mQTLs database (http://www.mqtldb.org) (51) to identify potential catalogued genetic variants that have been associated with the DNA methylation of our sites of interest. Altogether, we identified 1330, 1374, 1329 and 1329 mQTLs where genetic variation was strongly associated with the loci cg01201512, cg26371957, cg14911689 and cg26654770, respectively (**Additional File_mQTLs**).

Next, we sought to investigate whether the relation between CpG methylation that we found associated with PSC-UC was reflected in differential gene expression of this particular gene, through measuring *NINJ2* transcripts using quantitative PCR. However, we found no differential expression of *NINJ2* between PSC-UC and UC patients (**Figure 3E**), suggesting that the observed difference in methylation does not affect the expression of *NINJ2*.

Additional classification analysis on PSC-UC and HC samples yielded a predictive model with an area under the receiver-operator characteristic curve (AUROC) of 0.83. The CpG positions observed in this classification did not overlap with the CpGs as compared to the predictive model on PSC-UC and UC (**Supplementary Table 3**).

### Peripheral Blood Cell Distribution Is Comparable Between Patients With PSC-UC and UC

**Figure 4:**
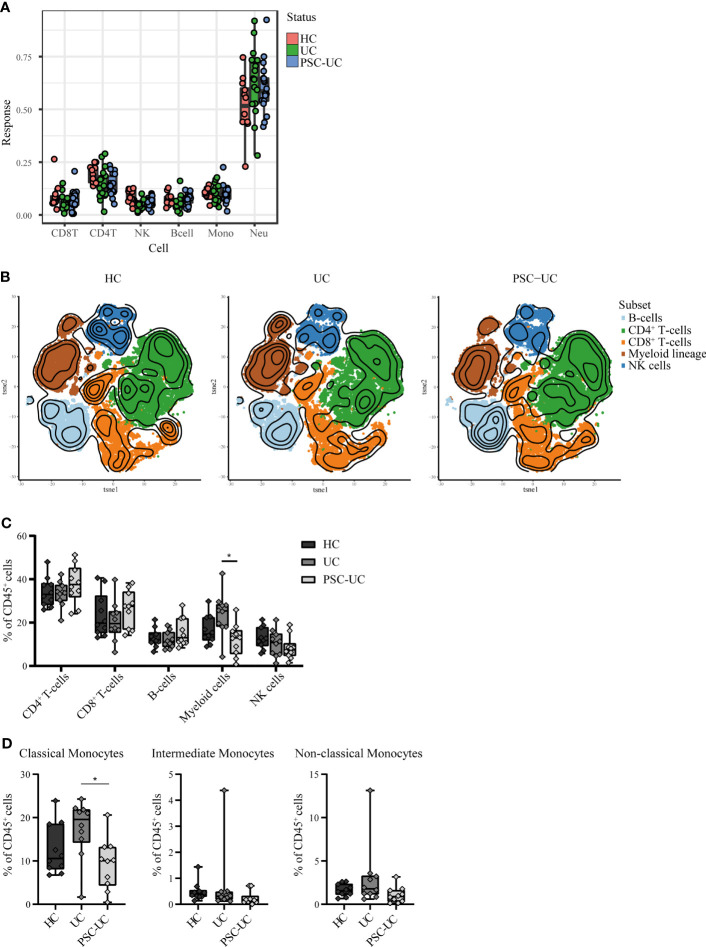
High-dimensional mass cytometry (cyTOF) analysis reveals that peripheral blood cell distribution is comparable between patients with PSC-UC, UC and HC. **(A)** Estimated cell proportions as derived from Houseman algorithm cell mixture deconvolution from DNA methylation data of PSC-UC (blue), UC (green) and HC (red). **(B)** The lineages CD4^+^T-cells, CD8^+^T-cells, B-cells, myeloid cells and NK-cells were clustered for PSC-UC, UC and HC and color-coded visualized using vISNE. **(C)** Differences in frequencies of CD4^+^T-cells, CD8^+^T-cells, B-cells, Myeloid lineage and NK-cells as percentage of total CD45^+^cells in HC (n = 9), UC (n = 10) and PSC-UC (n = 10). **(D)** Differences in frequencies of classical monocytes, intermediate monocytes and non-classical monocytes percentage of total CD45^+^cells in HC (n = 9), UC (n = 10) and PSC-UC (n = 10). Statistical testing was performed using Kruskal Wallis with Dunn’s correction for multiple testing. A p-value < 0.05 was considered statistically significant (*p < 0.05).

Due to the epigenetic and hence cell-specific nature of DNA methylation, observed differences could be the result of differences in the cellular composition of peripheral blood between PSC-UC and UC patients (52, 53). We therefore estimated the cellular composition using the algorithm described in Houseman et al. (**Figure 4A**) (54). We found no evidence that any of the estimated lineages (CD4^+^ T-cells, CD8^+^ T-cells, B-cells, natural killer (NK)-cells, monocytes and neutrophils) were different when comparing PSC-UC with UC. By contrast, we observed a significantly lower abundance of NK cells in the PSC-UC and UC samples as compared to HC (p-value = 5.33E-04).

**Figure 5:**
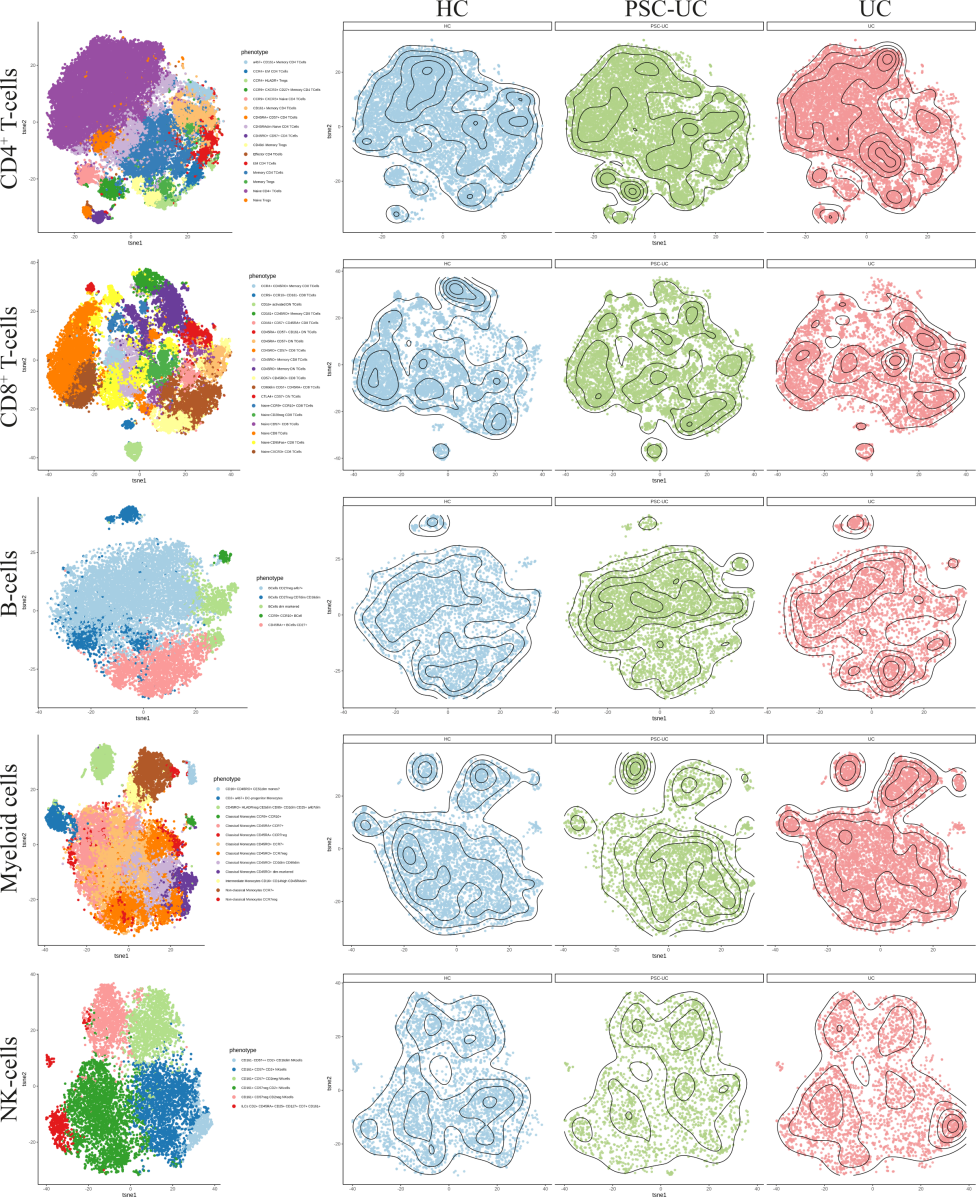
High dimensional mass cytometry (cyTOF) analysIs reveals that peripheral blood cell distribution is comparable between patients with PSC-UC, UC and HC. The lineages CD4^+^ T-cells, CD8^+^ T-cells, B-cells, myeloid cells and NK cells were clustered for PSC-UC, UC and HC and color coded visualized using vISNE, a visualization tool for high dimensional single cell data based on the t Distributed Stochastic Neighbor Embedding (t-SNE) algorithm.

As cellular estimates based on DNA methylation are currently limited in their resolution, we explored the peripheral blood mononuclear cellular distribution in more detail using mass cytometry. Blood samples were collected from a new cohort of PSC-UC (n = 10), UC (n = 10) and HC (n = 9). The median age at inclusion was 40, 58 and 30 years (p=0.022) (**Table 1**, cohort 2), for PSC-UC, UC and HC respectively. UC duration was significantly longer in patients with UC without PSC (median 22 years (IQR 14-34) compared to 9 years (IQR 5-19) in patients with PSC-UC (p=0.019). The majority of PSC-UC and UC patients used Mesalazine (90% in both groups) with only one patient with UC (without PSC) using a thiopurine. Different lineages were identified based on cellular phenotype and visualized in tSNE plots **(** **Figure 5**, **Figure 4B**). Akin to our observations made at the level of DNA methylation, the abundance of CD4^+^ T-cells, CD8^+^ T-cells and B-cells was not statistically different between PSC-UC, UC and HCs (**Figure 4C** and **Supplementary Figure 4**). Notably, we observed a similar trend in NK cell distribution as seen in the Houseman algorithm estimation. The myeloid lineage was significantly more abundant in patients with UC compared to patients with PSC-UC (p-value = 0.013, **Figure 4C**). Disentangling the myeloid lineage into the constituent monocyte subtypes revealed that this difference was predominantly observable in the classical monocyte (CD14^++^CD16^-^) population (**Figure 4D**). Within the classical monocyte populations, we particularly saw a decrease in CD45RA^+^CCR7^+^ classical monocytes and CD45RO^+^ CD2^DIM^ CD69^DIM^ classical monocytes among PSC patients compared to UC patients. Although the overall T-cell population did not differ between PSC-UC and UC, we observed distinct proportions of memory T-cells expressing CD161, a c-type lectin like-receptor expressed by NK cells, T helper 17 cells and mucosal invariant T (MAIT) cells (55) between patients with PSC-UC and UC (**Supplementary Figure 4**).

## Discussion

Distinguishing patients with UC and concomitant PSC from patients with UC could give more insights in disease pathophysiology of PSC and would be of clinical value for early diagnosis. In this explorative study, we performed genome-wide DNA methylation and mass cytometry analysis on whole peripheral blood from patients with PSC-UC, UC and HCs. We show that minor differences exist in the peripheral blood methylome when comparing male patients with PSC-UC to male patients with solely UC. Notwithstanding, classification analysis yielded a predictive model capable of distinguishing patients with PSC-UC and UC.

The overall lack of large-scale differences in methylation between the various groups corroborates the observations made by Moore et al., where the authors did not observe large global methylation changes in the peripheral blood of patients with PSC compared to healthy controls and between patients with PSC with and without IBD (15). While peripheral blood is a practical tissue for biomarker use due to its ease of access, it contains a heterogeneous population of cells, which might be less representative for disease features, such as the PSC-UC-associated phenotypes that manifest primarily in the liver and gut tissue. Subtle differences between PSC-UC, UC and HC may therefore remain hidden in the analysis performed.

Indeed mass cytometry analyses revealed subtle differences in blood cell composition across patient groups. While we demonstrated that the peripheral blood cell distribution was largely comparable between patients with PSC-UC and UC, the myeloid lineage, the monocyte CD14/CD16 subsets in particular, were more abundant in patients with UC compared to PSC-UC and HCs (56). As DNA methylation signal can vary between different cell types, observed differences might be reflective of changes in the underlying population (53).

Importantly, we could not confirm an increased DNA methylation age in PSC patients as was reported by Trauner et al. (16). The discrepancy between our observations might be related to their phenotype of interest, namely progression of fibrosis, for which we have no information of at the time of sampling. The fact that the median PSC duration was low in our cohort (5 years), might have influenced our accelerated age differences. Notwithstanding, we believe that the observed differences were likely influenced by the choice of normalization as Trauner et al. utilized quantile normalization, a method that presented one of the largest offset as compared to other normalization methods (57).

Classification analysis yielded a predictive model, which enables a distinction between peripheral blood of patients with PSC-UC and UC with an AUROC of 0.80, indicating that these two disease entities do have a distinct epigenetic architecture. The discrepancy between our DMP analysis and the classification analysis may be due to the non-linear relationships identified through gradient boosting. Whereas none of the predictive CpG-associated genes had been described within the context of PSC previously, two genes are associated to hepatocellular carcinoma, namely *CUL2* and *SOX6* (58–60). Moreover, both *CUL2* and *SOX6* are associated with colitis as well, while *SERPINB9* and *NINJ2* have been associated with colorectal cancer (61–64).

The four predictive CpG associated to *NINJ2* were found to be hypermethylated in PSC-UC compared to UC patients, which we validated through bisulfite sequencing. While the four CpGs presented a methylation pattern reminiscent of an underlying genetic variant (65), we did not identify any genetic variants through Sanger sequencing at the sites of interest. This does not eliminate the possibility that other genetic variants might have conferred the observed methylation signal, such as copy number variations (CNV), indels and more distal SNPs associated with differences in DNA methylation (51, 66, 67), which we were not able to pick up with the techniques utilized in the current study. With over a thousand catalogued mQTLs associated to the four *NINJ2*-associated predictive CpGs, the possibility is present that distal genetic variants may influence the methylation status of *NINJ2* (51).

The biological consequence of the observed difference in methylation of *NINJ2* remains to be established. The encoded Ninjurin 2 or nerve injury induced protein 2 (NINJ2) is an adhesion molecule expressed in neurons and glial cells and is involved in nerve regeneration (68). Notably, Ninjurin 1, a homologue of Ninjurin 2, as well as *NINJ2* are highly expressed in myeloid cells and peripheral leukocytes, suggesting a role in immune-mediated diseases as well (44, 69, 70). In vascular endothelial cells it was found that *NINJ2* regulates monocyte-adhesion as well as endothelial inflammation through the expression of pro-inflammatory cytokines such as IL-1β, TNF-α, IL-8, IL-6, ICAM-1 and E-selectin (70). Through mass cytometry we observed a diminished abundance of the myeloid population among the PSC-UC patients relative to the UC patients. The observed differences in myeloid cell population abundance may therefore be related to the observed difference in *NINJ2* methylation. However, while *NINJ2* expression was reportedly associated with DNA methylation in CD4^+^ T-cells (71), we observed no difference in *NINJ2* gene expression in the current cohort. This might be attributable to the differences in methylation being observed in the first intron rather than the promotor region. In summary, the role of *NINJ2* in relation to PSC-UC remains unclear.

Our study is not without its limitations: we only included male subjects to reduce variables and limit confounding in DNA methylation analysis. While having a same-sex cohort decreases variance, it makes the observations less translatable to the general PSC-IBD population. While previous EWAS have indicated that DNA methylation profiles differ between males and females (72), limited to no sex-associated difference were reported in PSC-associated EWAS (15, 16). One study did show an enrichment of differentially methylated CpG sites located on chromosome X in patients with PSC-IBD and PSC without IBD compared to controls but did not make a comparison with the methylomes of patients with PSC alone (15). While the majority of the patients with PSC are male, we acknowledge that further validation experiments in a larger and more heterogeneous population with both male and female subjects are necessary to properly ascertain the robustness of the predictive CpGs and the potential utility in the clinic in defining PSC-UC.

## Conclusions

Our study provides a novel approach of exploring DNA methylation analysis to differentiate patients with PSC-UC from patients with UC. Further validation in a different cohort has to confirm the biomarker potential of these methylation differences for early detection of PSC.

## Data Availability Statement

The datasets presented in this study can be found in online repositories. The names of the repository/repositories and accession number(s) can be found below: https://ega-archive.org/, EGAS00001005832.

## Ethics Statement

The studies involving human participants were reviewed and approved by Medical Ethics Committee at the Amsterdam UMC, University of Amsterdam, Ref. nrs: METC 06–267/E and METC 2018-050. The patients/participants provided their written informed consent to participate in this study.

## Author Contributions

Concept and design of the study: MK, CP, and WJ. MK assembled the patient cohorts, prepared samples for the DNA methylation array and performed the mass cytometry. PvH performed RNA isolation. JV, JGV, and MW analyzed the mass cytometry data. IH and TH performed Sanger sequencing and next generation sequencing. ALY and EL analyzed and visualized the results acquired from the HumanMethylation EPIC BeadChip array. EL performed machine learning. MK, IH, and ALY drafted the manuscript. PH, TH, CP, and WJ supervised the study. All authors read and approved the final manuscript.

## Funding

ALY was funded by the European Union’s Horizon 2020 research and innovation program under Grant Agreement No. ITN-2014-EID-641665. ALY and IH were funded by the Helmsley charitable trust. 

## Conflict of Interest

Author EL was employed by Horaizon BV.

The remaining authors declare that the research was conducted in the absence of any commercial or financial relationships that could be construed as a potential conflict of interest.

## Publisher’s Note

All claims expressed in this article are solely those of the authors and do not necessarily represent those of their affiliated organizations, or those of the publisher, the editors and the reviewers. Any product that may be evaluated in this article, or claim that may be made by its manufacturer, is not guaranteed or endorsed by the publisher.
