# Supplementary material for: Epigenetic Signatures Discriminate Patients With Primary Sclerosing Cholangitis and Ulcerative Colitis From Patients With Ulcerative Colitis
Source: Front Immunol. 2022 Mar 16;13:840935. doi: 10.3389/fimmu.2022.840935 (PMC8965896; doi:10.3389/fimmu.2022.840935)
Supplement: Supplementary file 1 [file DataSheet_1.pdf]

Supplementary Material

Supplementary Methods

1000 ng DNA was used as input in 8 ul total (H2O added), 2 ul of primermix (5 mM of each primer) and 10 ul of DreamTaq® Green PCR Master Mix (2X) was added to each sample. The following PCR program was used:

1. 95°C, 3 minutes
2. 98°C, 20 seconds
3. 62°C, 20 seconds
4. 72°C, 1 minute
5. step 2 – step 4 repeated 34 times
6. 72°C, 5 minutes
7. 4°C, ∞

After the PCR amplification samples were loaded on an ethidium bromide-stained 1% agarose gel and checked for the presence of a PCR product with the right size, followed by PCR clean-up with the Agencourt AMPure PCR purification kit (Beckman Coulter) in ratio 1:1. Cleaned up PCR products were used for Sanger sequencing. BigDye® Terminator v1.1 cycle sequencing was performed before Sanger sequencing, this cycle sequencing was a simple method in which successive rounds of denaturation, annealing and extension in a thermal cycler results in the incorporation of fluorescent dye labelled terminators into the DNA extension products. 10 ng DNA input in a total of 6,5 ul (H2O added) was used, 3,5 ul of mastermix was added (1 ul 0,5 mM of either reverse or forward NINJ2 primer, 0,5 ul of BigDye Terminator v1.1 cycle and 2 ul of 5x BigDye® Terminator sequencing buffer V1.1). The samples were ran in the PCR with the following program:

1. 96˚C, 60 seconds
2. 96˚C, 30 seconds
3. 50˚C, 10 seconds
4. 60˚C, 4 minutes
5. step 2- step 4 30x repeated

Supplementary Tables

Supplementary Table 1 – Top 10 differentially methylated regions when comparing PSC-UC with UC

Chromosome	Start	End	Width	No. cpgs	Min FDR	Stouffer	Max betafc	Mean betafc	Overlapping promoters
Chr 15	94651923	94652513	591	3	0.0236	0.899857	-0.03557	-0.02017	CTD-2643K12.3
Chr 16	56486263	56486336	74	3	0.010073	0.899857	-0.03981	-0.03013	NUDT21 OGFOD1
Chr 2	42275672	42276442	771	3	0.071437	0.899857	0.023197	0.015015	PKDCC
Chr 2	1.72E+08	1.72E+08	473	3	0.008439	0.899857	0.033163	0.024358	SP5
Chr 20	47364640	47365070	431	3	0.004625	0.899857	0.106079	0.084602	NA
Chr 20	61640931	61641454	524	3	0.016971	0.899857	0.084884	0.071537	RP11-305P22.9
Chr 22	40389805	40390264	460	3	0.015927	0.899857	0.037399	0.0208	FAM83F
Chr 4	1.22E+08	1.22E+08	324	3	0.010769	0.899857	0.057458	0.046863	TNIP3
Chr 7	1.17E+08	1.17E+08	46	3	0.015845	0.899857	0.045642	0.040346	ST7

Top 10 differentially methylated regions in comparison between patients with PSC-UC and UC. Minfdr, minimal FDR adjusted p-value; Stouffer, metric of interest for defining statistically significant DMRs; maxbetafc, maximum difference in beta values within this region; meanbetafc, mean difference in beta values within this region; NA, not applicable.

Supplementary Table 2 – Methylation status of probes associated with PSC-associated genes

	PSC-UC vs UC				PSC-UC vs HC			
	**NSig**	**Total**	**Prop**	**pval**	**NSig**	**Total**	**Prop**	**pval**
BACH2	23	137	0.202	**0.010**	8	137	0.062	**0.042**
ASAP2	18	112	0.191	**0.027**	5	112	0.047	0.165
DDIT4	9	58	0.184	0.052	7	58	0.137	**0.024**
CBX5	4	48	0.091	0.104	0	48	0.000	0.509
FOXP1	15	295	0.054	0.157	14	295	0.050	**0.018**
NFKB1	7	69	0.113	0.168	3	69	0.045	0.406
ITGAE	5	111	0.047	0.169	4	111	0.037	0.085
RSPO3	3	32	0.103	0.227	2	32	0.067	0.163
CFLAR	8	160	0.053	0.266	2	160	0.013	0.942
ITGB1	3	32	0.103	0.285	2	32	0.067	**0.028**
TCF4	11	162	0.073	0.296	5	162	0.032	0.831
GPR35	1	26	0.040	0.327	0	26	0.000	0.620
CCL25	0	12	0.000	0.343	0	12	0.000	0.144
PIGF	5	77	0.069	0.346	2	77	0.027	0.663
CCR10	0	18	0.000	0.376	0	18	0.000	0.747
FUBP1	4	27	0.174	0.392	1	27	0.038	0.192
STAT3	4	54	0.080	0.403	2	54	0.038	0.396
CDH1	2	56	0.037	0.421	1	56	0.018	0.769
CLEC16A	12	179	0.072	0.423	5	179	0.029	0.463
CXCL12	1	27	0.038	0.456	0	27	0.000	0.605
IL2RA	2	38	0.056	0.460	3	38	0.086	0.095
ITGA4	5	58	0.094	0.474	1	58	0.018	0.140
CCL28	1	29	0.036	0.482	1	29	0.036	0.382
SIK2	6	48	0.143	0.491	1	48	0.021	0.452
EML4	3	46	0.070	0.491	0	46	0.000	0.092
RBM5	4	43	0.103	0.500	0	43	0.000	0.704
CXCL16	2	47	0.044	0.612	4	47	0.093	0.051
MS4A4A	1	15	0.071	0.615	0	15	0.000	0.595
ITGB7	2	45	0.047	0.635	4	45	0.098	0.112
HDAC7	1	63	0.016	0.638	6	63	0.105	0.123
MADCAM1	1	17	0.063	0.654	1	17	0.063	0.379
CCDC88B	2	47	0.044	0.659	0	47	0.000	0.461
CD226	3	28	0.120	0.661	2	28	0.077	0.202
PSMG1	4	54	0.080	0.666	5	54	0.102	0.134
TNFAIP6	0	11	0.000	0.681	1	11	0.100	**0.036**
TENT2	0	2	0.000	0.704	0	2	0.000	0.259
ADM	1	38	0.027	0.774	0	38	0.000	0.321
PPIA	0	18	0.000	0.774	0	18	0.000	0.550
WTAP	1	30	0.034	0.811	2	30	0.071	0.248

CCR9	1	14	0.077	0.816	0	14	0.000	0.117
KIAA0146	2	44	0.048	0.822	2	44	0.048	0.110
PPP3CB	0	34	0.000	0.826	1	34	0.030	0.624
RIC8B	1	36	0.029	0.833	1	36	0.029	0.326
SMAD5	1	25	0.042	0.836	0	25	0.000	0.119
N4BP2L2	1	54	0.019	0.839	1	54	0.019	0.828
JAK3	1	45	0.023	0.849	4	45	0.098	0.063
UBASH3A	2	30	0.071	0.851	6	30	0.250	**0.006**
PCGF3	5	122	0.043	0.924	2	122	0.017	0.485
IREB2	1	33	0.031	0.958	2	33	0.065	0.455
SOCS3	0	39	0.000	0.960	4	39	0.114	**0.039**
MST1	0	5	0.000	0.987	0	5	0.000	0.215
CD28	0	17	0.000	0.998	3	17	0.214	**0.007**

Supplementary Table 3 – Regions/DMPs associated with PSC-UC upon classification analysis on PSC-UC and HC samples

**Location**	**Associated gene**	**Chromosome**	**Difference in methylation in PSC-UC vs UC**	**Feature Importance**
cg23899408	*HOOK2*	Chr 19	Hypermethylation	4.031324
cg16397968	*PFKP*	Chr 10	Hypomethylation	3.166667
cg22715629	*CNOT1*	Chr 16	Hypermethylation	3.145012
cg11738485	*HOOK2*	Chr 19	Hypermethylation	2.812812
cg16361921		Chr 5	Hypomethylation	2.078962
cg05995465	*HDAC4*	Chr 2	Hypermethylation	1.91791
cg03997626	*SIAH3*	Chr 13	Hypomethylation	1.467478
cg02168270		Chr 8	Hypomethylation	1.437908
cg18136963		Chr 6	Hypomethylation	1.416403
cg06417478	*HOOK2*	Chr 19	Hypermethylation	1.187516
cg08919780	*CDK13*	Chr 7	Hypermethylation	0.96423
cg04657146	*HOOK2*	Chr 19	Hypermethylation	0.883303
cg09352518	*ZNF714*	Chr 19	Hypomethylation	0.880487
cg17404449	*LOC100133091*	Chr 7	Hypermethylation	0.810458
cg13830619		Chr 12	Hypomethylation	0.802466
cg07037055		Chr 13	Hypermethylated	0.754085
cg23923019	*ABCC5-AS1.ABCC5*	Chr 3	Hypermethylated	0.64244
cg07796016	*LCE1C*	Chr 1	Hypermethylated	0.602941
cg22888160		Chr 1	Hypermethylated	0.578918
cg06864789		Chr 6	Hypomethylated	0.556649
cg10993865	*CARS2*	Chr 13	Hypermethylated	0.490196
cg02693227	*ALG1L2*	Chr 3	Hypomethylated	0.478548
cg13774807	*TEK*	Chr 9	Hypermethylated	0.392125
cg02533724		Chr 10	Hypomethylated	0.277778
cg25755428	*MRI1*	Chr 19	Hypomethylated	0.272321

Supplementary Table 4 – Primer sequences

Gene	Technique	Forward sequence	Reverse sequence
NINJ2	Sanger Sequencing	CCCTCATCAGCCTCTCTCTG	CGTTGTTGAGCTGGTTGAGT
		GATCCCAAGGGCTTGACTCC	GAAAAAGAGCACTCTTCAGCCC
		TCGTCGGCAGCGTCAGATGTGT ATAAGAGACAGCTCAGGTGAG TAAATTCGCAGA	GTCTCGTGGGCTCGGAGATGTG TATAAGAGACAGTTTGGAGACG GAGGTGGATA
NINJ2	Bisulfite PCR-Seq	CGTCGGCAGCGTCAGATGTGTA TAAGAGACAGAGGTTGTGATA GGGGTTATTA	GTCTCGTGGGCTCGGAGATGTG TATAAGAGACAGACCTATATAA TACAAATCCCCAAAAA
NINJ2	qPCR	QT00088200 (Qiagen)	
GAPDH	qPCR	GTCAGTGGTGGACCTGACCT	TGAGCTTGACAAAGTGGTCG
HPRT	qPCR	CCTGGCGTCGTGATTAGTGAT	AGACGTTCAGTCCTGTCCATAA

Supplementary Table 5 – Antibody Panel Mass Cytometry

Antibodies pre-fixation:

Target	Metal	Source	Purpose
CD194 (CCR4)	158Gd	Fluidigm	T and B lymphocytes, basophils, monocytes and NK cells
CD195 (CCR5)	144Nd	Fluidigm	T lymphocytes and monocytes
CD183 (CXCR3)	156Gd	Fluidigm	Chemokine receptor, T lymphocytes
α4β7	171Yb	Takeda	Intestinal homing T lymphocytes
CCR9	168Er	Fluidigm	Intestinal homing T lymphocytes
CCR10	148Nd	R&D	Intestinal homing T lymphocytes
Cisplatin	194–195Pt	Fluidigm	Live/dead discrimination

Antibodies nuclear staining:

Target	Metal	Source	Purpose
CD152 (CTLA-4)	161Dy	Fluidigm	Co inhibitory molecule
CES-1	175Lu	Thermo Scientific	Myeloid cells

Antibodies post-fixation:

Target	Metal	Source	Purpose
CD45	89Y	Fluidigm	All leukocytes
CD49d	141Pr	Fluidigm	Integrin alpha subunit.
CD11a	142Nd	Fluidigm	Integrin alpha L chain
CD5	143Nd	Fluidigm	Activated lymphocytes
CD4	145Nd	Fluidigm	T helper lymphocytes
CD8a	146Nd	Fluidigm	Cytoxic T lymphocytes
CD7	147Sm	Fluidigm	T lymphocytes
CD25 (IL-2R)	149Sm	Fluidigm	Activated T lymphocytes, regulatory T cells
CD134 (OX40)	150Nd	Fluidigm	Co-stimulatory molecule
CD2	151Eu	Fluidigm	T lymphocytes, NK cells
CD95/Fas	152Sm	Fluidigm	Apoptosis
TIM-3	153Eu	Fluidigm	Co inhibitory molecule
CD14	154Sm	Fluidigm	Monocytes
CD279 (PD-1)	155Gd	Fluidigm	Activated lymphocytes, immune checkpoint
CD197 (CCR7)	159Tb	Fluidigm	Activated lymphocytes, homing to sec. lymphoid organs
CD28	160Gd	Fluidigm	Co-stimulatory molecule, activated T lymphocytes
CD69	162Dy	Fluidigm	Activated lymphocytes
CD161	164Dy	Fluidigm	NK cells
CD45RO	165Ho	Fluidigm	All leukocytes
CD44	166Er	Fluidigm	Activated lymphocytes
CD27	167Er	Fluidigm	Activated lymphocytes
CD45RA	169Tm	Fluidigm	All leukocytes
CD3	170Er	Fluidigm	T lymphocytes
CD57	172Yb	Fluidigm	NK cells
CD137/4-1BB	173Yb	Fluidigm	Macrophages, activated B cells, and dendritic cells
HLA-DR	174Yb	Fluidigm	Antigen presenting cells
CD127 (IL-7Ra)	176Yb	Fluidigm	Memory and effector T cells, immature B cell proliferation
CD16	209Bi	Fluidigm	Monocytes
Barcodes	103–110Pd	Fluidigm	Staining standardization and doublet discrimination
Iridium	191–193Ir	Fluidigm	Cell identification

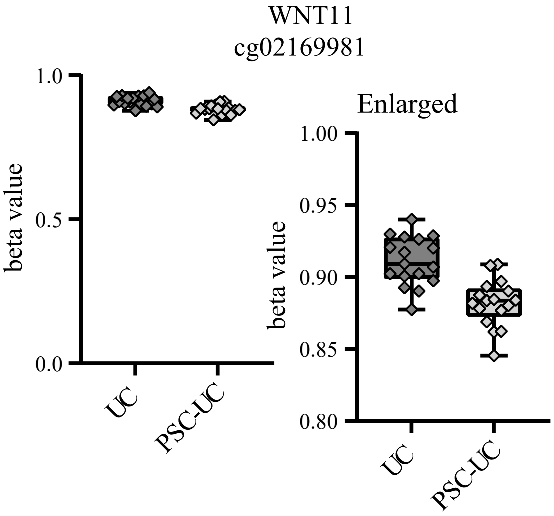

Supplementary Figure 1. WNT11 contains a differentially methylated position in patients with PSC-UC compared to patients with UC. A: Dot boxplot with enlarged representation for the CpG cg02169981 associated to WNT11 with visualization of the percentage methylation (beta value) on the Y axis for PSC-UC and UC.

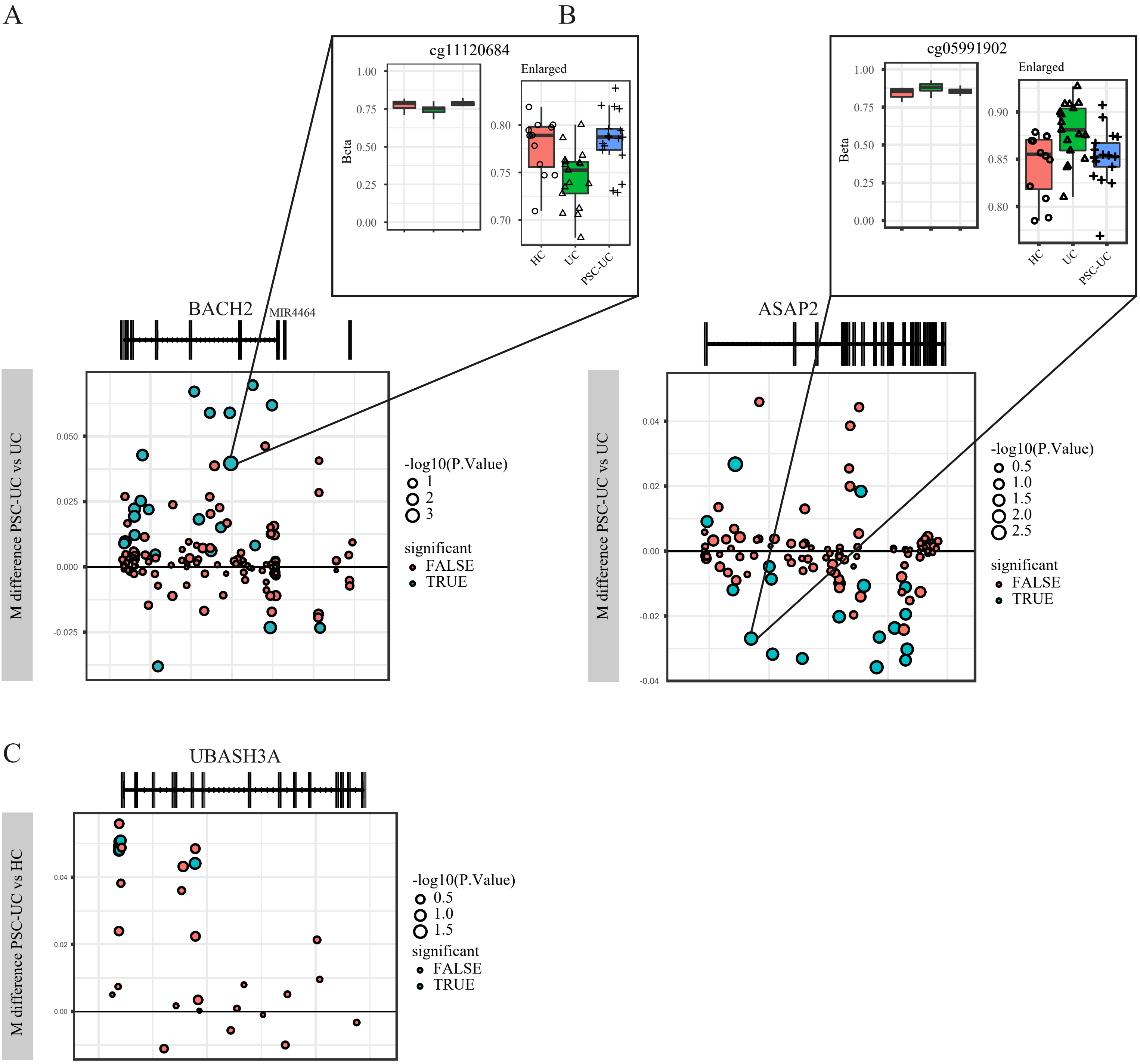

Supplementary Figure 2. BACH2 and ASAP2 contain hypothesis driven differentially methylated positions in patients with PSC-UC compared to patients with UC. Visualization of the loess smoothed mean percentage methylation difference PSC-UC vs UC per probe across the gene representation for BACH2 and ASAP2. Sizes of the dots represent the log10(p value). A: Enlarged dot boxplots for the significant CpG cg11120684 associated to BACH2. B: Enlarged dot boxplots for the significant CpG cg05991902 associated to ASAP2. C: Visualization of the loess smoothed mean percentage methylation difference PSC-UC vs HC per probe across the gene representation for UBASH3A.

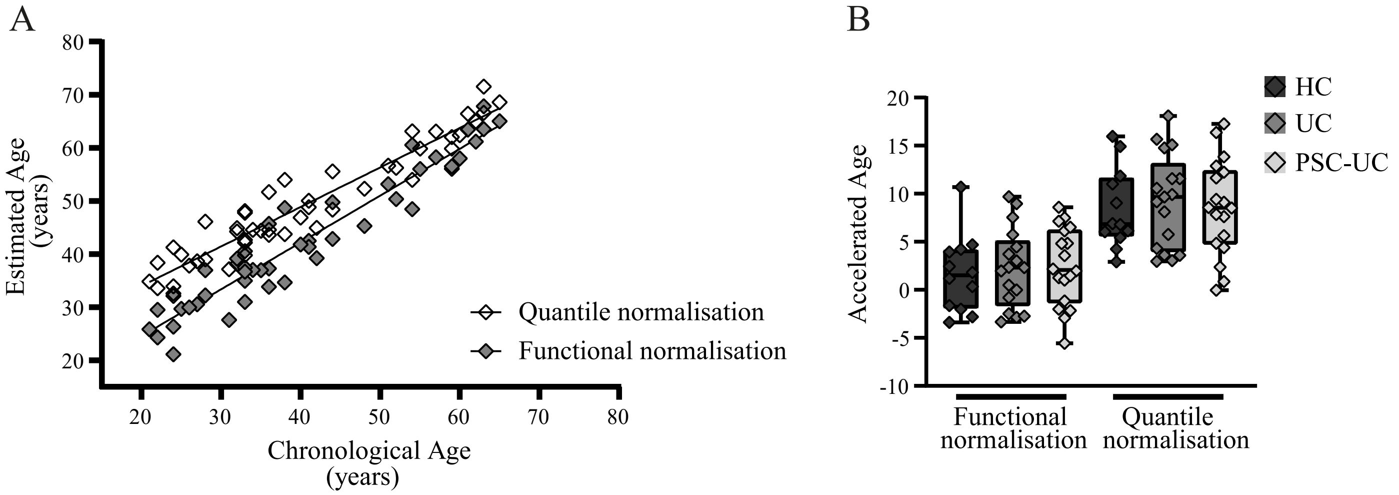

Supplementary Figure 3. Comparison of normalisation methods for determination of age acceleration. A: Correlation between chronogical age and estimated age based on DNA methylation using both quantile and functional normalization methods. B: Comparison of accelerated age (difference between chronogical age and estimated age) between patients with PSC-UC, UC and HC with both functional and quantile normalisation.

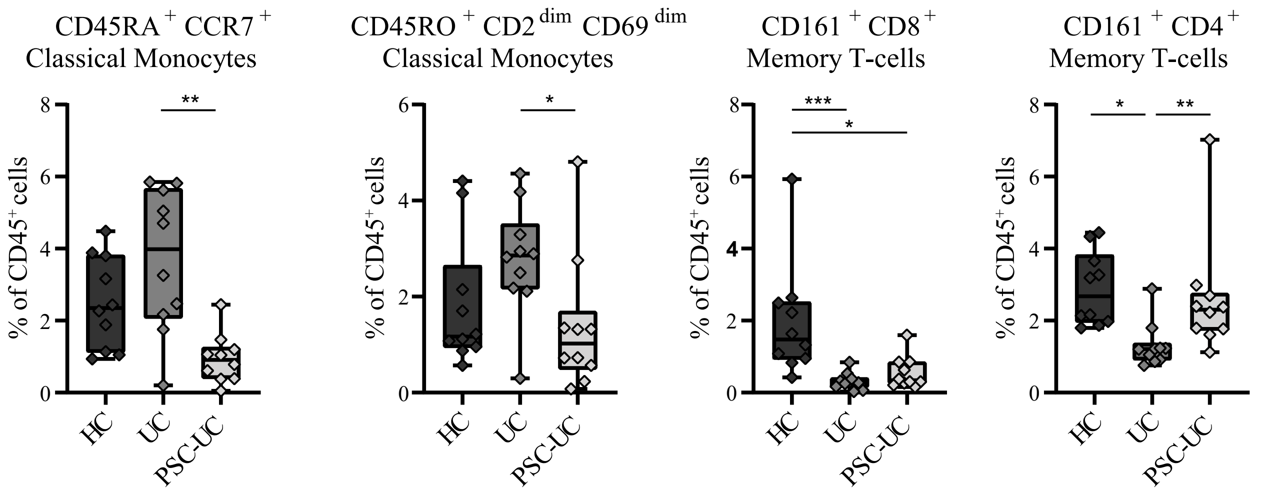

Supplementary Figure 4. Peripheral blood cell distribution of differential immune cell populations between PSC-UC, UC and HCs. Immune cell phenotype frequencies measured with cyTOF per patient cohort plotted as a fraction of total CD45+ cells. Statistical testing was performed using Kruskal Wallis with Dunn's correction for multiple testing. A p-value <0.05 was considered statistically significant (*p<0.05, ** p<0.01, ***< 0.001).

```
AGCTCTCAGGTGAGTAAATTCGCAGATGCCCACGCATGTCTGGAACATACCCCAGGGGTCATTAGAGAC
TGCCCAGCAGAGGCGGGAGGATCTTCCGTTTGCTTTTCCTTCAGAACGTTTGTCCGGAGAGCATCCTGT
TCTGTTATATGCATATCCACCTCCGTCTCCAAATTACTAGGTTGAGCACAGTGCTCAGTGCCTGGCATT
GAGTTAGAAATAGAAAATGAGCTGGCGAGTCCCGGCCCGAGGCTGTGACAGGGGCCATTACTGAGCCCA
CTCCCCACCAACCTTTCTGCGGTTTAACTGACTCTCCTCCTTCCCCTCCAGATCCCAAGGGCTTGACTC
CTACCTCCTCTAAGGCATTTTGAAAAGTTTGCAAATGCACCCTTGATCCTAGCACGTACTTAAGAAGCG
GCTCCCCCCTAGAACGTCAGGCTTGAAGCTGACCCCTTCTTCCTCCTCCCGCGCCATTTGGGTCTGTGC
GCAGGGCCGGAAGCCTGCTGGGGAGGGGCACGAGGCCGGGCTGGGGCCTCTCCGGAGGGAGCCCGCCGC
CCCGCCCAGGGCGCACGCACCCAGCCTCTGAGGGAGGAAGCTGAATGGGGCCAGAGGCTGGCCCTCTTT
GGGGATTTGCATTATACAGGCTGTATTTAGAAGCAGCGGCGGGGAGCGGTGGCTCACGCCTGTGATCCC
AGCATTTTGGGGGGCCGAGGCGGGCAGATCACCTGAGGCCAGGAGTTCGAGACCAGCCTGTCAACATGG
TGAAACTCCGTCTGTGCTAAAAATACAAAAAAAATTAGCCAGGCGTGGTGGCCGGAGCCTGCAGTCCCA
GCTACTCGGGAGGCTGAGGCAGGAGAATCGCTTGAACCCGGGAGATGGAGCTTGCAGTGAGCCGAGATC
ACACCACTGCACTCCAGCCTGGGCAACTCAGCGAGACTCCGTCTCAAAAAACAAAACAAAACAAAACAA
AACAAAAAACCAAGAAAAAGAGAATAAAAAAAAATAGAAGCAGCTTTCTTGCTGATAGAAAGCAATGAC
CCTCTCTGCTATAATTACAACTGAAGAGTCACTTTCTTTCAAAAACTCTATTTTCAGATGTTTACCACT
TTCTTTAAAAAGGAAAAAAATTTGCCTCAAAGGCAGGCAGTCCCCAGGGCATTTCTGGTCTCCTGGGGA
AGGAACCTAAGAGCGGTTTGGTAAAATTCCATTTTGCTATCTTGGCGTTACCTAAGGGCAAGAACTGAG
GTGGGGCTTTGGTGACAGCAGGGGCTGAAGAGTGCTCTTTTTCATCATCCAGGGTGTC
```

Supplementary Figure 5. Reference sequence of NINJ2. Position chr12:739,280 - 740,338 (human genome build 19). In yellow marked the CpG sites of interest with predictive value for distinguishing PSC-UC and UC: cg01201512 (chr12:740,338), cg14911689 (chr12:739,980) cg26371957 (chr12:739,280) and cg26654770 (chr12:740,100).
